# Endocannabinoid Modulation of Cortical Up-States and NREM Sleep

**DOI:** 10.1371/journal.pone.0088672

**Published:** 2014-02-10

**Authors:** Matthew J. Pava, Carolina R. den Hartog, Carlos Blanco-Centurion, Priyattam J. Shiromani, John J. Woodward

**Affiliations:** 1 Department of Neurosciences and Center for Drug and Alcohol Programs, Medical University of South Carolina, Charleston, South Carolina, United States of America; 2 Department of Psychiatry & Behavioral Sciences, Medical University of South Carolina, Charleston, South Carolina, United States of America; 3 Ralph H. Johnson VA Medical Center, Charleston, South Carolina, United States of America; Hôpital du Sacré-Coeur de Montréal, Canada

## Abstract

Up-/down-state transitions are a form of network activity observed when sensory input into the cortex is diminished such as during non-REM sleep. Up-states emerge from coordinated signaling between glutamatergic and GABAergic synapses and are modulated by systems that affect the balance between inhibition and excitation. We hypothesized that the endocannabinoid (EC) system, a neuromodulatory system intrinsic to the cortical microcircuitry, is an important regulator of up-states and sleep. To test this hypothesis, up-states were recorded from layer V/VI pyramidal neurons in organotypic cultures of wild-type or CB1R knockout (KO) mouse prefrontal cortex. Activation of the cannabinoid 1 receptor (CB1) with exogenous agonists or by blocking metabolism of endocannabinoids, anandamide or 2-arachidonoyl glycerol, increased up-state amplitude and facilitated action potential discharge during up-states. The CB1 agonist also produced a layer II/III-selective reduction in synaptic GABAergic signaling that may underlie its effects on up-state amplitude and spiking. Application of CB1 antagonists revealed that an endogenous EC tone regulates up-state duration. Paradoxically, the duration of up-states in CB1 KO cultures was increased suggesting that chronic absence of EC signaling alters cortical activity. Consistent with increased cortical excitability, CB1 KO mice exhibited increased wakefulness as a result of reduced NREM sleep and NREM bout duration. Under baseline conditions, NREM delta (0.5–4 Hz) power was not different in CB1 KO mice, but during recovery from forced sleep deprivation, KO mice had reduced NREM delta power and increased sleep fragmentation. Overall, these findings demonstrate that the EC system actively regulates cortical up-states and important features of NREM sleep such as its duration and low frequency cortical oscillations.

## Introduction

Low frequency oscillations in electrical activity called slow-waves (0.5–4 Hz) become the dominant pattern of cortical activity when sensory input to cortical networks is reduced, for instance during deep-stage non-REM (NREM) sleep, anesthesia, and in *cerveau isolé* preparations [1]. Simultaneous electrocorticogram (ECoG) and intracellular recordings in anesthetized cats demonstrate that slow-waves emerge from membrane potential bistability of cortical neurons [2] characterized by transitions between a hyperpolarized, quiescent “down-state” and a depolarized “up-state” that is crowned with fast post-synaptic potentials (PSPs).

Up-states reflect robust signaling at both glutamatergic and GABAergic synapses, and modulation of AMPA-, NMDA-, or GABA-mediated currents significantly alters the initiation and maintenance of the these events [3]. For example, up-states are modulated by monoaminergic inputs arising from midbrain and brainstem structures [4]–[7]. Nonetheless, organotypic cortical cultures lacking monoaminergic inputs still actively generate up-states [7]–[9] suggesting that extra-cortical neuromodulators are not essential for this form of network activity. However, it is not known whether activity within and between pyramidal neurons (PNs) and interneurons in the cortical microcircuitry may act synergistically with intrinsic neuromodulatory systems to regulate network activity.

Endocannabinoids (ECs) are a class of atypical neurotransmitters synthesized and released from the post-synaptic membrane of cortical PNs during periods of enhanced cellular activity such as during up-states [10]. Therefore ECs could be considered as an intrinsic neuromodulatory system. ECs bind to the presynaptic cannabinoid 1 (CB1) receptor [11] that mediates most of the physiological effects of cannabinoids in the CNS [12], [13]. In the cortex, activation of CB1 decreases release of both GABA and glutamate [14] suggesting this local neuromodulatory system may tune network activity by regulating both excitatory and inhibitory neurotransmission within local cortical circuits.

To examine if ECs may regulate the excitatory and inhibitory inputs to the cortical neurons, we recorded up-states from layer V/VI pyramidal neurons in organotypic cultures of prefrontal cortex (PFC) prepared from wild-type (*wt*) and CB1 knockout (KO) mice. The results from pharmacological studies revealed that activation of CB1 enhances up-state amplitude, while an EC tone modulates up-state duration, likely via CB1-dependent inhibition of GABAergic transmission onto layer II/III PNs. Yet EC signaling is not essential for up-states since cultures prepared from CB1 KO mice still displayed this form of network activity. In fact, up-state duration in KO cultures was longer than in controls suggesting that compensatory processes had developed that resulted in a more active cortical network. Consistent with this observation, ECoG recordings from CB1 KO mice found that these animals sleep less and, following sleep deprivation, exhibit reduced power of slow-wave oscillations associated with up-state activity *in vivo*. Our data suggest that the EC system is an important modulatory system that regulates up-states *in vitro* and sleep-wake states *in vivo*.

## Materials and Methods

### Ethical Approval

Housing and treatment of all animals used in this study conformed to the *Guide for the Care and Use of Laboratory Animals* [15], and all experimental protocols were approved by the Medical University of South Carolina Institutional Animal Care and Use Committee (animal protocols 2825 and 2371). In all, 94 mice were used as subjects for the experiments reported in this manuscript.

### Subjects

Breeding pairs of wild-type C57BL6/J mice, originally purchased from the Jackson Laboratories (Bar Harbor, ME) were maintained in breeding colonies at the Medical University of South Carolina. Heterozygous breeding pairs of CB1 null mutant mice (CB1 KO mice; [16]) on a C57BL6/J background were obtained as a kind gift from Dr. David Lovinger (NIAAA). Mice from this colony were used for the slice culture studies (PN 1–5) and the ECoG recordings (∼12 weeks old).

### Cortical Organotypic Cultures

Slice cultures of neonatal mouse cerebral cortex were prepared as previously described with some modifications [7]. Postnatal day 1–5 mouse pups were deeply anesthetized by placing them in an ice water bath for at least 2 min prior to decapitation. Brains were extracted and immediately submerged in ice-cold sterile-filtered HEPES-buffered, sucrose dissection solution containing (in mM): 200 sucrose, 1.9 KCl, 6 MgCl_2_, 0.5 CaCl_2_, 10 glucose, 0.4 ascorbic acid, 25 HEPES, pH 7.3. Coronal sections (400 µm) containing the anterior cingulate (ACC), prelimbic and infralimbic regions of the prefrontal cortex were prepared on a vibrating microtome under sterile conditions in a biosafety hood equipped with laminar flow. Slices representing the right and left hemispheres of each section were transferred to Millicell-CM 0.4 µm biopore membrane inserts (Millipore Corp., Billerica, MA) in six-well culture plates containing 1 mL pre-incubated high-serum media. This media contained 50% basal medium Eagle, 25% Earle's balanced salt solution, 25% heat-inactivated horse serum (HIHS), 5.9 mg/mL glucose, 25 mM HEPES, 100 µg/mL streptomycin, and 0.235 mM Glutamax. Slices were arranged on the membranes so that their dorsal surfaces were aligned and their medial surfaces were touching.

Cultures were maintained in an incubator at 37°C and a 5% CO_2_ atmosphere with partial media exchanges every 2–3 days. Beginning at 3 days *in vitro* (DIV), high-serum media was replaced with media containing 5% HIHS. At 14 DIV, culture media was supplemented with 20 µM 5-fluoro-2-deoxyuridine to prevent glial overgrowth. All recordings from cultures were made after 14 DIV to allow recovery from slicing and for the cortical network to mature.

### Whole-Cell Electrophysiology

On the day of recording, cultures were removed from the incubator, and the membrane immediately surrounding the culture was cut from the rest of the insert while taking care not to damage the tissue. The culture was then submerged in a recording chamber perfused at 2 mL/min with ACSF containing (in mM): 125 NaCl, 2.5 KCl, 1.25 NaH_2_PO_4_, 1.3 MgCl_2_, 2.0 CaCl_2_, 0.4 ascorbic acid, 10 glucose, 25 NaHCO_3_, 0.05% bovine serum albumin (BSA) and continuously bubbled with carbogen gas (95% O_2_/5% CO_2_). Bath temperature was maintained at 32.0±0.5°C using a heated recording chamber and an in-line flow-through heater controlled by a thermistor-coupled TC-342B temperature controller (Warner Instruments, Hampden, CT). For current-clamp experiments, patch-pipettes (1.5 mm×1.1 mm; 1.8–3.5 MΩ) were filled with internal recording solution containing (in mM): 130 K-gluconate, 10 KCl, 2 MgCl_2_, 0.1 EGTA, 10 HEPES, 2 NaATP, 0.3 NaGTP, pH 7.3. For voltage-clamp recordings, patch-pipettes were filled with a solution containing (in mM): 140 CsCl, 2 MgCl_2_, 0.1 EGTA, 10 HEPES, 2 NaATP, 0.3 NaGTP, 5 QX-314, pH 7.3. Whole-cell patch-clamp recordings were made from visually identified pyramidal neurons (PN) in the region of cultured cortex corresponding to the ACC. Neurons were imaged using a Zeiss FS2 microscope (Oberkochen, Germany) equipped with an infrared video camera and Dodt gradient contrast optics. For all recordings, gigaohm seals were obtained in voltage-clamp mode using an Axoclamp 700A amplifier (Molecular Devices, Sunnyvale, CA). For current-clamp experiments, the amplifier mode was switched following breakthrough. Pipette access resistance (5–25 MΩ) was monitored throughout experiments and cells showing a significant deviation in access resistance (>25%) were not used for analysis. Square-wave electrical stimuli (0.1 msec) used to evoke up-states or post-synaptic currents (PSCs) were generated by a stimulus isolation unit and delivered to the tissue via a concentric bipolar electrode placed in the cortical tissue. For current clamp recordings, up-states were recorded in layer V/VI neurons and were evoked with a 750 µA pulse delivered at 0.033 Hz to the lateral aspect of the cortex (frontal association cortex or secondary motor cortex), distal to the recording site. Up-state amplitude was measured as the average membrane potential (V_m_) during the plateau of the up-state minus the average V_m_ during the down-state immediately preceding stimulation. Up-state duration was measured as the time from stimulation to the inflection point where the V_m_ settled back to the down-state potential. For voltage clamp recordings of evoked PSCs the stimuli (75–200 µA) were delivered to cortical layer II/III proximal to the recording electrode at 0.05 Hz.

GABA_A_-mediated inhibitory PSCs (IPSCs) were recorded from neurons held at −70 mV in ACSF supplemented with (*R*,S)-amino-5-phosphonovaleric acid (DL-APV; 100 µM), 6,7-dinitroquinoxaline-2,3-dione (DNQX; 20 µM), and (2*S*)-3-[[(1*S*)-1-(3,4-Dichlorophenyl)ethyl]amino-2-hydroxypropyl] (phenylmethyl) phosphinic acid (CGP-55845; 1 µM) to block NMDA-, AMPA-, and GABA_B_- mediated responses, respectively. NMDA-mediated excitatory PSCs (EPSCs) were recorded from cells held at +40 mV in ACSF supplemented with picrotoxin (100 µM) and DNQX and were used to monitor excitatory signaling. Recordings of monosynaptic AMPA-mediated EPSCs could not be reliably obtained from the organotypic cultures used in this study likely due to the highly recurrent synaptic structure of these cultures that results in large amplitude polysynaptic responses following stimulation of neurons held at −70 mV. Data for all experiments were filtered at 4 kHz and acquired at 10 kHz. The amplifier output was digitized by an ITC-16 interface (Instrutech, Port Washington, NY) controlled by AxoGraphX software (AxoGraph Scientific, Sydney, Australia) running on a Macintosh G4 (Apple Inc., Cupertino, CA).

All cannabinoid drugs (WIN 55,212-2 (WIN), AM251, AM281, NESS0327, tetrahydrolipstatin (THL), URB597, and JZL184) were dissolved in DMSO and subsequently diluted to working concentrations in ASCF containing 0.5% BSA as a carrier. In all cases, final DMSO concentrations were below 0.1%. In time course experiments involving hour-long recordings, sham treated groups were included to control for the effects of exposure to DMSO, BSA, and repeated stimulation. Sham treated tissue was exposed to similar experimental regimen of stimulation and the presence of DMSO/BSA in the ACSF with the exception that no drugs were applied.

### Electrocorticography, Sleep Scoring, and Power Spectral Analysis

Mice were housed in a controlled reverse light/dark cycle (lights on from 6:00 P.M. to 6:00 A.M.) with food and water provided *ad libitum*. Supracortical sleep recording electrodes were implanted as previously described [17]. CB1 KO and wild-type mice (12 weeks or older) were implanted with sleep recording electrodes under isoflurane anesthesia (2%). The ECoG was recorded from four stainless steel screws placed in the skull to sit on surface of the cortex. Two screws were inserted 2 mm from either side of the sagittal suture and 3 mm ahead of bregma. The other two were inserted 3 mm on either side of the sagittal suture and 3 mm behind bregma. Electromyogram (EMG) data was monitored using two stainless-steel flexible multiwire electrodes deeply inserted into the nuchal muscles. After 1 week of recovery from surgery, mice were connected to lightweight recording cables and given at least 10 days to adapt to tethering. ECoG and EMG were recorded for a consecutive 48-h baseline period. Following baseline recordings, subjects were gently deprived of all sleep (total sleep deprivation; TSD) during the first 6 hr of the light photoperiod by periodically changing cage bedding and introducing novel nesting material [18]. At the end of TSD, mice were returned to their home cage and allowed to sleep undisturbed. EEG recordings were obtained throughout the enforced wakefulness and 12 hr of post- sleep deprivation.

The ECoG signal was recorded from two contralateral screws (frontal-occipital cortices) and was filtered at 100 Hz (low-pass filter) and 0.3 Hz (high-pass filter) using a Grass Instruments model 15ES1 polygraph (Quincy, MA). Data was continuously sampled at 128 Hz by a 486 Intel microprocessor computer with an analog-to-digital board (National Instruments, Austin, TX). EMG activity was acquired using the same polygraph and filtered between 1 kHz and 100 Hz. The ECoG signal was scored manually on a computer (ICELUS Software; Dr. Mark Opp, University of Michigan, Ann Arbor, MI) in 12 s epochs as wake, NREM sleep and REM sleep. Wake was characterized by a low-amplitude-fast frequency ECoG with high EMG integral activity. NREM sleep consisted of high-amplitude slow waves with low EMG tone, and REM sleep was identified by presence of regular theta activity coupled with low EMG tone. The amount of time spent in wake, NREM and REM was determined for each hour for the 48 h recording and averaged into 3-h bins. Wake, NREM, and REM bout duration and number was manually calculated considering a minimum criteria for acceptance of >1 epoch of duration. Calculation of delta (0.5–4 Hz) power during NREM sleep was done automatically by the scoring software (ICELUS) using only artifact-free episodes. To accommodate for large variance in ECoG power between subjects, the sum of NREM delta power for each hour of recording was normalized to the total power (0.5–20 Hz) observed in the power spectrum for that hour using the following equation:
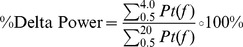
where *P_t_* is the power spectrum for that hour.

### Statistical Analysis

Analyses of sleep/wake data and the results of power spectral analysis were performed in PASW18 (IBM, New York, NY). A hierarchical linear model (HLM) was used for hypothesis testing in sleep studies and in analysis of power spectral data. In these analyses, time bins were nested within photoperiod and represent within-subjects factors whereas genotype was a between-subjects factor. A first-order autoregressive variance-covariance matrix was used in the model to account for unequal variances between time bins. Model effects and pair-wise comparisons are reported using an F statistic. Bonferroni corrected *p*-values were used to determine significance for all multiple comparisons tests. Statistical analysis of whole-cell electrophysiology data was performed using Prism 4 (GraphPad Software, San Diego, CA). A Bonferroni correction was used for *p*-values resulting from post-hoc ANOVA tests. For measures of PSC inhibition by WIN:
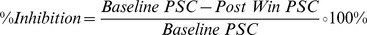
For all analyses α = 0.05.

## Results

### Endocannabinoids modulate up-states via activation of the CB1 receptor

**Figure 1:**
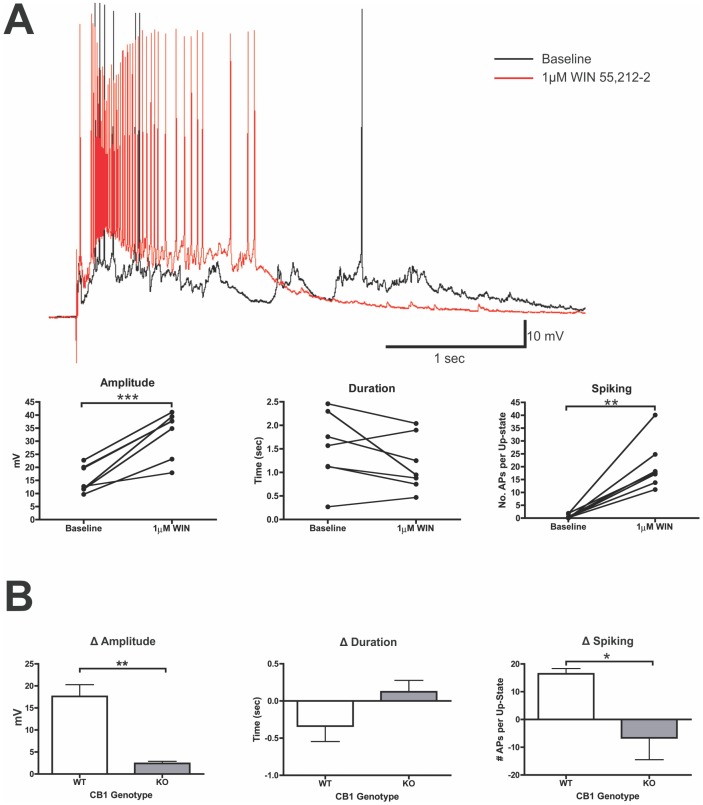
The CB1 agonist WIN 55,212-2 enhances up-state amplitude and spiking. A, example traces from the same neuron before and after application of WIN (1 µM) and summary data from these experiments. In graphs, connected points represent data from the same neuron. B, comparison of the change in up-state parameters following WIN treatment in *wt* and CB1 KO cultures. Bars represent mean ± SEM of difference scores for each measure of up-states (*N* = 7 cells). Symbols: *p<0.05, **p<0.01, and ***p<0.001.

To test whether CB1 receptors regulate up-state parameters, the CB1 agonist WIN (1 µM) was bath applied to cultures following baseline recordings of up-states (Fig. 1A). After a 10 min application of WIN, there was a significant enhancement of up-state amplitude (paired t-test, t(6) = 6.57, *p*<0.001) and number of action potentials per up-state (spiking) (t(6) = 5.59, *p* = 0.0014). Treatment with WIN did not have a significant effect on up-state duration (t(6) = 1.16, *p* = 0.154). To confirm the CB1-dependence of the WIN effect, the change in up-state parameters before and after WIN application was compared between *wt* cultures and those prepared from CB1 null-mutant mice. The effects of WIN on up-state amplitude and spiking were largely CB1-dependent as the WIN-induced change in up-state amplitude (t-test, t(12) = 4.00, *p* = 0.0018) and spiking (Welch's t-test, t(5.573) = 2.85, *p* = 0.032) was substantially attenuated in cultures prepared from KO mice as compared to the effects observed in *wt* cultures (Fig. 1B).

**Figure 2:**
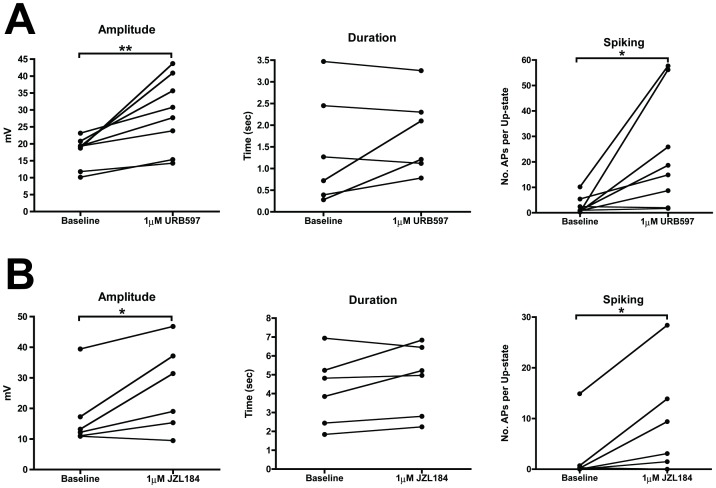
Selectively increasing either AEA or 2-AG by inhibiting FAAH or MAGL, respectively, increases up-state amplitude and spiking. A, effect of FAAH inhibition with URB597 (1 µM) on up-state parameters. B, effect of MAGL inhibition with JZL184 (1 µM) on up-state parameters. Connected points represent data from the same the cell during baseline and in the presence of the drug. Symbols: *p<0.05 and **p<0.01.

The results with WIN demonstrate that activation of the CB1 receptor modulates the amplitude of up-states, but the question remains if EC transmitters can modulate up-states. Anandamide (AEA) and 2-arachidonylglycerol (2-AG) are the two most well studied EC transmitters and have separate pathways for synthesis and inactivation. AEA synthesis occurs via several different pathways [19] while the enzyme fatty acid amide hydrolase (FAAH; [20]) is primarily responsible for its inactivation. 2-AG is synthesized via a rate-limiting step catalyzed by diacylglycerol lipase (DAGL), and is inactivated through monoacylglycerol lipase (MAGL)-mediated hydrolysis. To determine if endogenous ECs in slice cultures alter up-state parameters, we bath applied inhibitors of the major enzymes responsible for EC inactivation (Fig. 2). A 10 min bath perfusion with the FAAH inhibitor URB597 (1 µM) significantly increased up-state amplitude (paired t-test, t(7) = 3.79, *p* = 0.0068) and spiking (t(7) = 2.75, *p* = 0.029) but failed to alter duration (t(7) = 1.57, *p* = 0.161; Fig. 2A). Similarly, treatment with the selective monoacylglycerol lipase (MAGL) inhibitor JZL184 (1 µM; [21]) produced a significant increase in up-state amplitude (paired t-test, t(5) = 2.73, *p* = 0.041) and spiking (t(5) = 2.71, *p* = 0.042) with no effect on duration (t(5) = 1.77, *p* = 0.138; Fig. 2B). Thus, these treatments recapitulated the effects of WIN on up-state parameters and suggest that ECs are synthesized *in situ* and significantly alter network physiology when their concentrations are enhanced.

**Figure 3:**
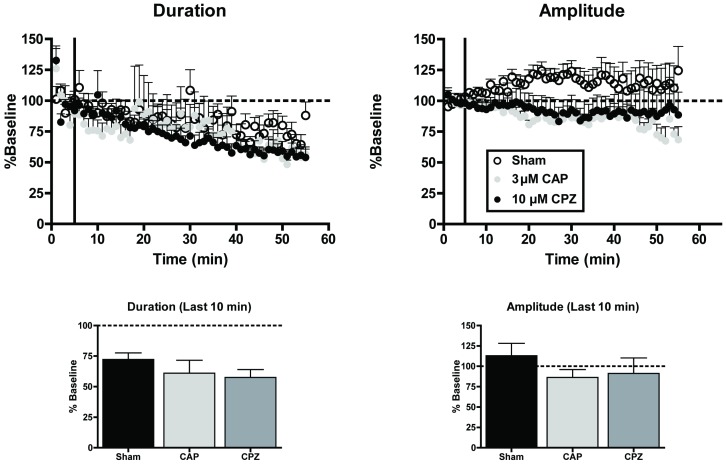
Modulation of TRPV1 receptor function does not alter up-states. Top graphs: time course of measures of up-state duration and amplitude in cultures treated with sham conditions, the TRPV1 agonist capsaicin (CAP; 3 µM), or the TRPV1 antagonist capsazepine (CPZ; 10 µM). Bottom graphs: summary data from the three treatment conditions binned over the last 10 min and normalized to their respective baselines. Bars represent mean ± SEM.

In addition to being a partial agonist for CB1 [22], [23], AEA also activates transient receptor potential cation channel subfamily V member 1 (TRPV1) receptors [24], ion channels that can induce a long-term depression at inhibitory [25] and excitatory [26] synapses. To determine whether AEA-mediated TRPV1 activation modulates network activity, up-states were measured in the presence of either the TRPV1 agonist capsaicin (CAP; 3 µM; Fig. 3) or the TRPV1 antagonist capsazepine (CPZ; 10 µM). These doses were chosen based on commonly used concentrations used in slice physiology experiments [25], [26]. After 50 min of bath application, neither CAP nor CPZ altered up-state duration relative to sham treated controls (one-way ANOVA, F(2, 21) = 1.50, *p* = 0.247). There were also no significant effects of these drugs on up-state amplitude (F(2, 19) = 0.67, *p* = 0.516) suggesting that, under the conditions used in this study, TRPV1 receptors do not regulate cortical up-states.

### Differential laminar distribution of CB1-mediated inhibition of glutamatergic and GABAergic signaling

**Figure 4:**
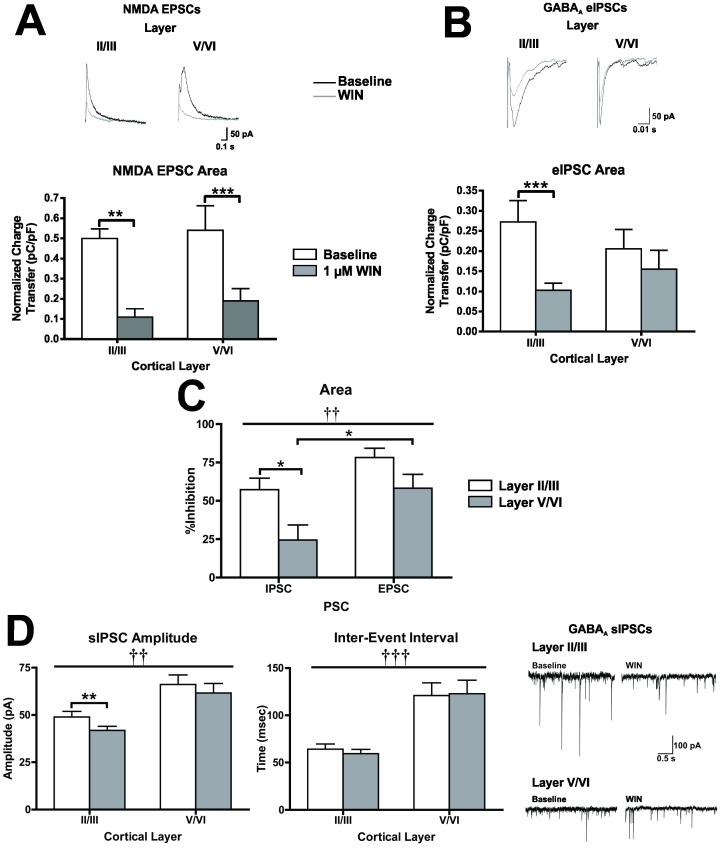
Laminar and synapse specific effects of CB1 activation with 1 µM WIN in organotypic cultures of PFC. A, Effect of WIN on evoked NMDA EPSCs recorded from layer II/III and layer V/VI PNs (*N* = 9). B, Effect of WIN on evoked (top graphs) and spontaneous (bottom graphs) GABA_A_ IPSCs recorded from layer II/III and layer V/VI pyramidal neurons (*N* = 8–11). In panels A and B, white bars represent baseline and grey bars represent PSCs in the presence of WIN. Traces to the right of panels A and B represent example traces from these experiments. For evoked PSCs, black traces represent baseline and grey traces represent PSCs in the presence of WIN. Significant main effects of cortical layer are denoted by a dagger (†) and significant pair-wise comparisons between baseline and WIN are indicated by an asterisks (*). C, comparison of the inhibitory effects of WIN between PSC type and across cortical layers (*N* = 8–12). White bars represent data from layer II/III PNs and grey bars represent recordings from layer V/VI PNs. Significant main effects of PSC type are denoted by a dagger (†) and significant post-hoc comparisons are indicated by an asterisks (*). For all graphs bars represent mean ± SEM. For all symbols conferring statistical significance: single symbol *p*<0.05, double symbol *p*<0.01, triple symbol *p*<0.001.

Activation of CB1 decreases neurotransmitter release at both glutamatergic [27] and GABAergic [28], [29] terminals. In somatosensory and auditory cortices, the effects of CB1 activation on GABAergic transmission prevail under physiological conditions producing a net disinhibition of cortical PNs [30], [31]. To determine the effects of CB1 activation on glutamatergic neurotransmission, NMDA EPSCs were recorded from PNs in layer II/III and layer V/VI before and after the application of WIN (1 µM; 10 min; Fig. 4A). There was no interaction between cortical layer and WIN treatment (two-way repeated measures ANOVA, F(1, 13) = 0.132, *p* = 0.723). However, there was a main effect of WIN to reduce EPSC area (F(1, 13) = 44.71, *p*<0.0001), and EPSCs were significantly reduced by WIN application in recordings from both layer II/III (pair-wise comparison, t(13) = 5.16, *p* = 0.0004) and layer V/VI neurons (t(13) = 4.33, *p* = 0.0016).

In a complementary set of experiments, the effects of CB1 activation (1 µM WIN; 10 min) on evoked and spontaneous IPSCs (IPSC and sIPSC, respectively) on layer II/III and layer V/VI neurons were compared (Fig. 4B & 4D). There was no interaction between cortical layer and WIN treatment (two-way repeated measures ANOVA, F(1, 15) = 4.29, *p* = 0.056), but there was a main effect of WIN to reduce IPSC area (F(1, 15) = 14.68, *p* = 0.0016). A within-group planned comparisons found that IPSC area was reduced in recordings from layer II/III neurons (t(15) = 4.60, *p*<0.001) but not layer V/VI (t(15) = 1.15, *p* = 0.538). Data from recordings of sIPSCs (Fig. 4D) confirmed the layer specific effects of CB1 activation. Overall, sIPSCs recorded from layer V/VI neurons were of higher amplitude than those recorded from layer II/III cells (main effect of cortical layer, two-way repeated measures ANOVA, F(1, 19) = 11.94, *p* = 0.0026). There was also a main effect of CB1 activation (F(1, 19) = 15.29, *p*<0.001), but this effect was layer specific as WIN treatment only reduced sIPSC amplitude in layer II/III neurons (pair-wise comparison, t(19) = 3.47, *p* = 0.0051). The sIPSC inter-event interval (IEI) was significantly greater in recordings from layer V/VI neurons (main effect of cortical layer, two-way repeated measures ANOVA, F(1, 19) = 21.54, *p*<0.001), but there was no effect of WIN (F(1, 19) = 0.23, *p* = 0.636) on sIPSC IEI.

In order to compare the effects of CB1 activation on glutamatergic and GABAergic synapses across cortical layers, the percent of inhibition was calculated for evoked currents, (Fig. 4C). WIN-induced inhibition of PSCs differed significantly between PSC type (main effect of PSC, two-way ANOVA, F(1, 34) = 11.2, *p* = 0.002) and cortical layer (main effect of layer, two-way ANOVA, F(1, 34) = 10.47, *p* = 0.0027). Specifically, activation of CB1 produced less inhibition of IPSCs in layer V/VI than EPSCs within the same layer (pair-wise comparison, t(34) = 2.80, *p* = 0.017), and CB1-mediated inhibition of IPSCs in layer II/III was greater than in deep-layer cells (t(34) = 2.90, *p* = 0.013). These data support the hypothesis that GABAergic synapses onto layer II/III PNs are more sensitive to the effects of CB1 activation than GABA synapses on layer V/VI PNs. Likewise our results indicate that glutamategic synapses in different layers show similar sensitivity to activation of CB1.

### Antagonists of CB1 block the manifestation of neocortical up-states

**Figure 5:**
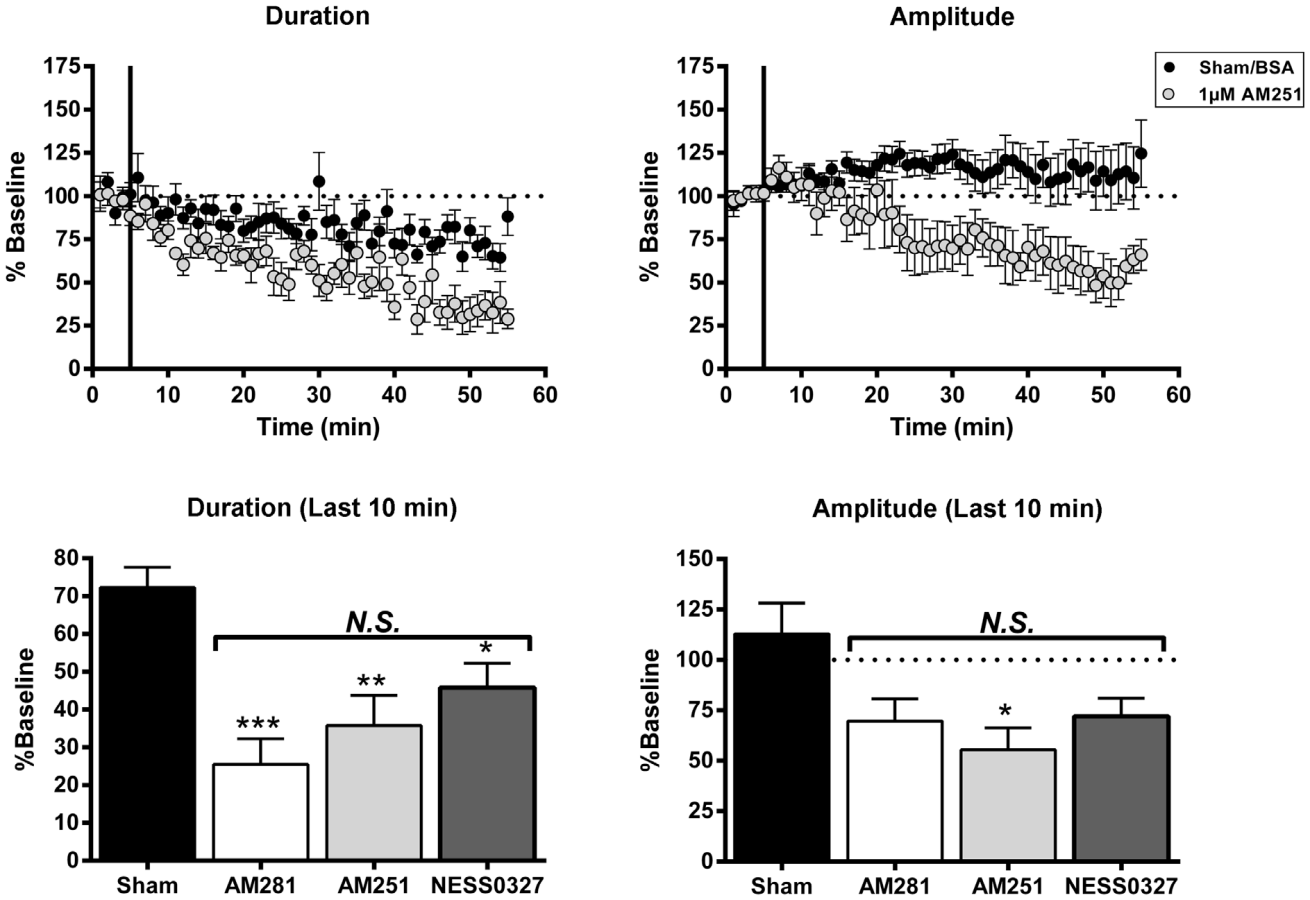
CB1 antagonists reduce up-state amplitude and duration. Top graphs: time course of measures of up-state duration and amplitude in cultures treated with sham conditions or the CB1 inverse agonist AM251 (1 µM). Points represent group means ± SEM. Bottom graphs: summary data from the four treatment conditions (sham, 1 µM AM281, 1 µM AM 251, 0.1 µM NESS0327) binned over the last 10 min and normalized to their respective baselines (*N* = 6–10). Bars represent group means ± SEM. Asterisks indicate significant difference from the sham group, and *N.S.* indicates no significant differences between drug treated groups. Symbols: *p<0.05, **p<0.01, ***p<0.001.

**Figure 6:**
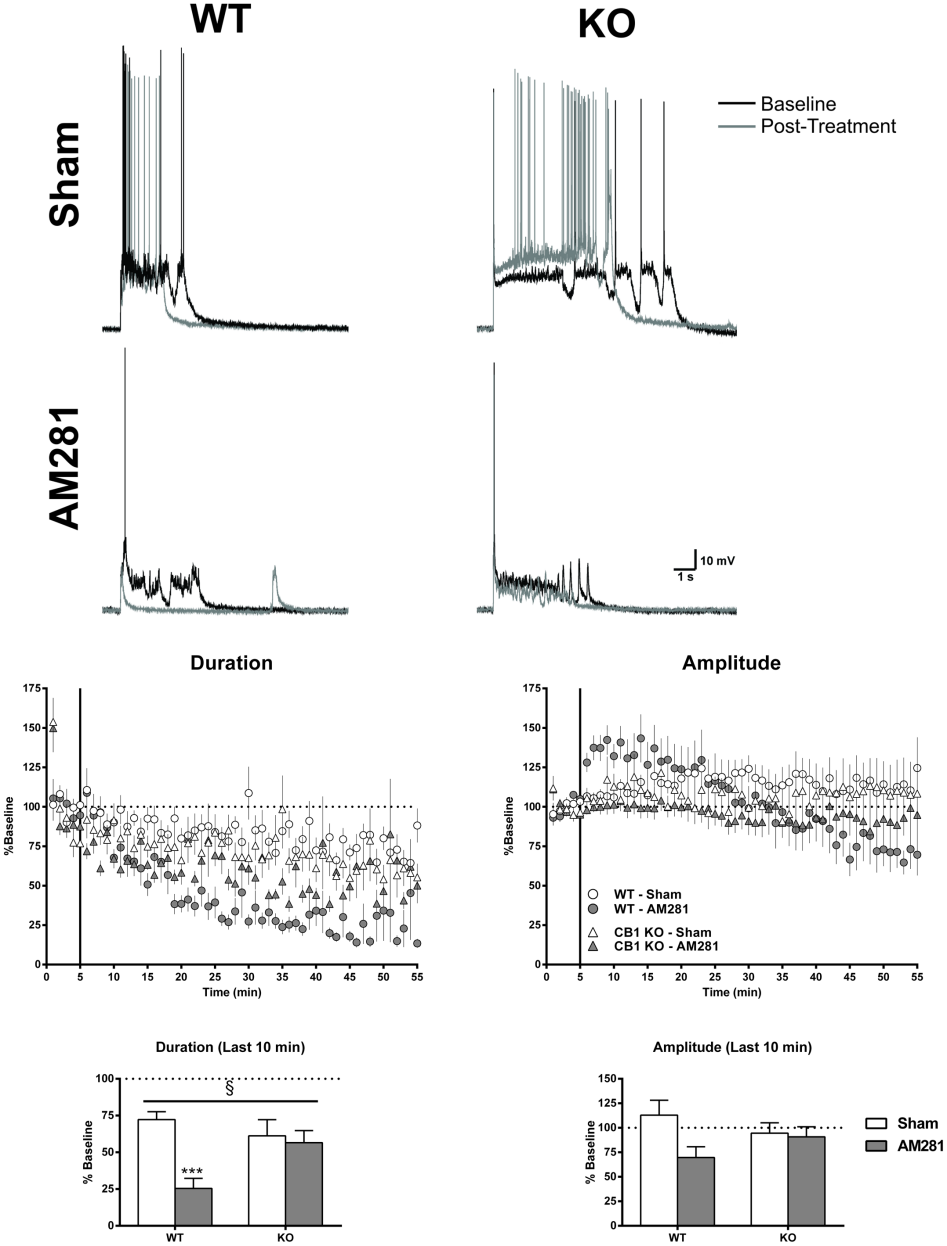
AM281 reduces up-state duration via a CB1-dependent mechanism. Top panel: example traces from each of the four groups showing baseline up-states (black traces) and those following 50 min of drug application (grey traces). Middle panel: time course of measures of up-state duration and amplitude in *wt* and CB1 KO cultures under sham conditions or treated with the CB1 inverse agonist AM281 (1 µM). Points are normalized to the pre-drug values for each genotype and represent group means ± SEM (*N* = 6–11) and. Bottom panel: summary data from the four groups binned over the last 10 min and normalized to their respective baselines (*N* = 8–10). Bars represent group means ± SEM. Symbols: § - significant interaction (*p*<0.05), *** - significant post-hoc comparison with *wt* sham (*p*<0.001).

As discussed above, inhibitors of FAAH and MAGL augmented up-state amplitude and spiking suggesting a role for ECs in regulating this form of cortical network activity. To investigate the necessity of EC/CB1 signaling in generating up-states, time course experiments were performed where up-states were evoked every 30 sec for 55 min in the presence of three structurally similar, well-characterized, selective antagonists of the CB1 receptor (Fig. 5). Specifically, the effects of 1 µM AM281 (K_i_ = 12 nM), 1 µM AM251 (K_i_ = 7.49 nM), and 0.1 µM NESS0327 (K_i_ = 0.35 pM) were compared. The doses used for AM281 and AM251 were chosen based on commonly used concentrations in slice physiology experiments [11], [31], [32]. Because NESS0327 is much more potent that the other two CB1 antagonists, an order of magnitude lower concentration was used. Application of any one of the CB1 antagonists significantly reduced up-state duration relative to up-states in sham treated control tissue (one-way ANOVA, F(3, 28) = 11.06, *p*<0.001) and in many cases, reduced up-states to a brief EPSP (see example trace from *wt* cultures treated with AM281 in Fig. 6). In pair-wise comparisons with control treated cultures (exposed to ACSF with the carrier molecule) up-state duration was similarly reduced by the inverse agonists AM281 (1 µM; t(28) = 5.51, *p*<0.001) and AM251 (1 µM; t(28) = 3.83, *p* = 0.004) and the neutral antagonist NESS0327 (0.1 µM; t(28) = 2.91; p = 0.042; [33]). The peak amplitude of up-states was also significantly reduced by the application of CB1 antagonists (one-way ANOVA, F(3, 26) = 3.90, *p* = 0.020); however, pair-wise comparisons with the sham group revealed this effect only reached significance for AM251-treated cultures (pair-wise comparisons, t(26) = 2.93, *p* = 0.042). Pair-wise comparisons between drug treatment groups found no significant differences (0.1235≤t(26)≤0.79, *p*>0.999).

Because AM281 produced the largest magnitude inhibition of up-state parameters (following treatment up-state duration was reduced to 25.5±5.3% of baseline, mean ± SEM), this drug was used to confirm that these effects were CB1-dependent. Cultures prepared from CB1 KO mice were exposed to sham treatment or AM281 (1 µM) while up-states were evoked, and these data were then compared to the AM281 and sham treated groups of *wt* cultures from the previous experiment (Fig. 6). To directly compare the effects of AM281 on up-state parameters, data were normalized to pre-drug baseline values for each genotype. AM281 produced only a slight reduction in up-state amplitude in the last 10 min of recording (69.7±11.0% Baseline, mean ± SEM), and a two-way comparison found no interaction (two-way ANOVA, F(1, 30) = 2.43, *p* = 0.13) or main effects of genotype (F(1, 30) = 0.011, *p* = 0.915) or treatment (F(1, 30) = 3.46, *p* = 0.073). However, a comparison of up-state duration from the last 10 min of recordings found a significant interaction between genotype and treatment (two-way ANOVA, F(1, 33) = 7.16, *p* = 0.0115) and a main effect of treatment (F(1, 33) = 10.70, *p* = 0.0025). As before, the duration of up-states in *wt* cultures treated with AM281 was significantly reduced compared to sham treated controls (pair-wise comparison, t(33) = 4.27, *p*<0.001), but there was no difference in the duration of up-states from sham or AM281 treated KO cultures (t(33) = 0.41, *p*>0.999).

**Figure 7:**
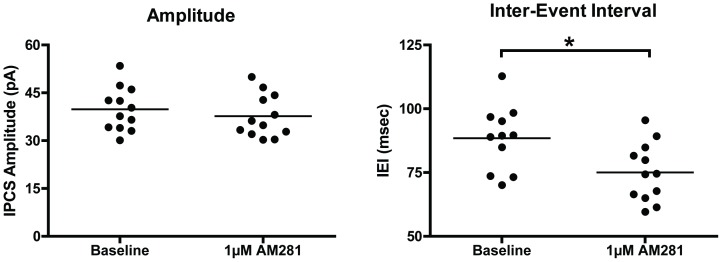
AM281 reduces the inter-event interval of GABA_A_ sIPSCs in layer II/III PNs. Each data point represents the average sIPSC amplitude or inter-event interval from one neuron (*N* = 11–13). Baseline data points represent different cells (but the same cultures) from those recorded after 1 hr of incubation with AM281 (1 µM). Horizontal lines represent group means. Asterisks (*) represents a significant group difference, *p*<0.05.

Up-states rely on a balance between synaptic activity at GABAergic and glutamatergic synapses and ongoing EC activation of CB1 decreases GABAergic signaling at layer II/III PNs as well as glutamate synapses throughout cortical layers. If EC tone critically maintains the appropriate ratio between glutamatergic and GABAergic signaling during up-states, then the diminished duration of up-states following application of CB1 antagonists might reflect enhanced GABAergic neurotransmission. To test this hypothesis, baseline recordings of sIPSCs from layer II/III PNs in *wt* cultures were compared to recordings from cultures incubated in AM281 (1 µM) for at least 1 hr (Fig. 7). The inter-event interval (IEI) of GABA_A_ sIPSCs was significantly reduced in AM281-treated cultures (t-test, t(21) = 2.67, *p* = 0.014), but there was no effect on sIPSC amplitude (t(22) = 0.79, *p* = 0.439) supporting the idea that blocking the activation of CB1 enhances GABAergic input onto layer II/III PNs.

**Figure 8:**
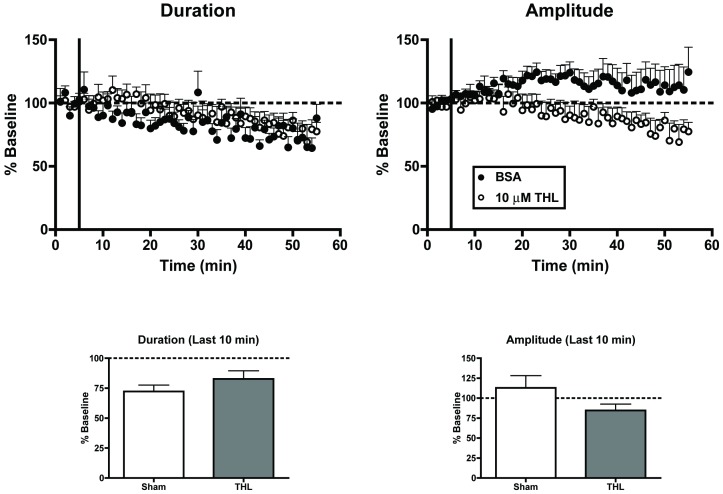
Blockade of 2-AG synthesis does not alter up-state parameters. Top graphs: time course of measures of up-state duration and amplitude in sham treated cultures and those treated with the DAGL inhibitor tetrahydrolipstatin (THL; 10 µM). Bottom graphs: summary data from the two treatment conditions binned over the last 10 min and normalized to their respective baselines. Bars represent mean ± SEM.

To identify whether AEA or 2-AG mediates the tonic regulation of up-states, the DAGL inhibitor, THL (10 µM), was applied to cultures while evoking up-states (Fig. 8). Compared to recordings from sham treated cultures, THL application failed to alter either up-state duration (t-test, t(16) = 1.23 *p* = 0.238) or amplitude (t(16) = 1.53, *p* = 0.147) suggesting that 2-AG is not responsible for mediating the EC tone regulating up-states.

### Altered up-state parameters in cortical cultures from CB1 KO mice

**Figure 9:**
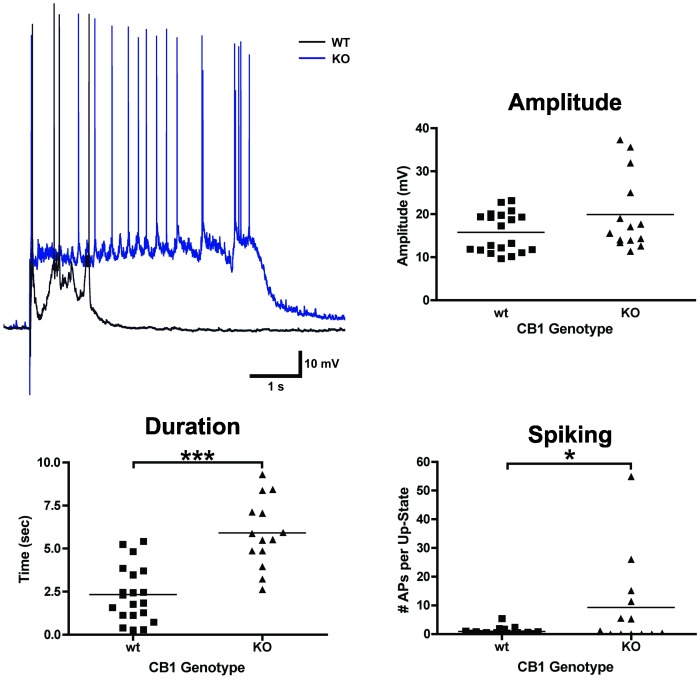
Up-states in CB1 KO cultures are longer than those recorded in *wt* cultures. Example traces in upper left-hand corner: black trace is a representative example of an up-state from a *wt* culture and blue trace is an example from a CB1 KO culture. Graphs show summary data from this series of experiments. Each data point represents the average measurement from a given neuron. Horizontal lines represent group means. Asterisks represent significant group differences. Symbols: *p<0.05 and ***p<0.001.

As noted earlier (Fig. 6), AM281 reduced up-state duration in wild-type cultures but did not affect duration of up-states in KO cultures. As a further test of the hypothesis that CB1 signaling is necessary for the manifestation of up-states, baseline measures of up-state parameters were compared between cultures prepared from *wt* and CB1 KO mice (Fig. 9). Up-state amplitude did not differ between genotypes (t-test, t(31) = 1.06, *p* = 0.3), but, in contrast to that found with CB1 antagonists, up-state duration in KO cultures was significantly enhanced as compared to control cultures (t(32) = 5.78, *p*<0.001). This was accompanied by an increase in the total number of spikes generated during the up-state (t(30) = 2.29, *p* = 0.029). These results demonstrate that signaling through CB1 is not necessary for the manifestation of the up-states, but in the chronic absence of CB1, cortical network activity is dysregulated resulting in increased excitability. Thus, signaling through CB1 is necessary for shaping normal patterns of cortical activity.

### Genetic Deletion of the CNR1 Gene Reduces NREM Sleep

**Figure 10:**
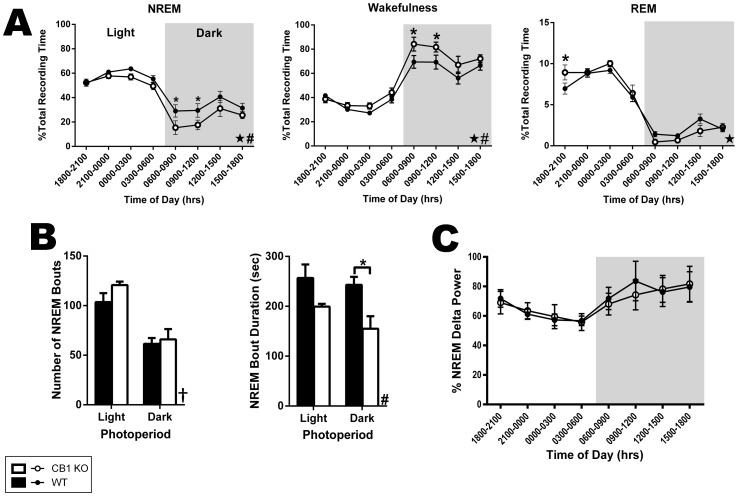
Genetic deletion of the CB1 receptor reduces the stability of NREM bouts by decreasing the time spent in NREM and increasing WK. A, Comparison between CB1 KO and *wt* mice (N = 6 in each group) in the time spent in NREM sleep, awake, and in REM sleep across the circadian period during baseline recordings. Data in each epoch were normalized to the total recording time to yield the percent of time spent in each state. B, Comparison of NREM architecture between genotypes. Graphs show data pertaining to the number and duration of NREM bouts. C, Comparison between genotypes for measures of delta power during NREM across the circadian cycle. Data represent the results of power spectral analysis of ECoG waveforms recorded during NREM sleep normalized to the total power across the power spectrum (0.5–20 Hz) for each time bin. For all graphs, black bars/circles represent data from *wt* mice, and white bars/circles represent data from CB1 KO mice. In time series graphs, the white background indicated the light photoperiod and the grey background denotes the dark photoperiod. Data represent the means ± SEM of each genotype within the specified time bin or photoperiod. For A and C, data were grouped in 3 hr bins. In B, each bar represents a 12 hr (one photoperiod) bin. Significant 3-way interactions between photoperiod, time of day, and genotype are denoted with stars (★). Significant main effects of genotype are denoted with pound signs (#). Significant main effects of photoperiod are denoted with a dagger (†). Significant pair-wise comparisons are denoted with asterisks (*).

Because the longer duration up-states observed in CB1 KO cultures reflect greater cortical activity, we hypothesized that sleep and delta frequency (0.5–4 Hz) oscillations would be reduced in CB1 KO mice. To test this, ECoG and EMG recordings were obtained from CB1 KO mice and *wt* littermates and scored for sleep-wake states. A comparison of time spent in the three main behavioral states (wake, NREM, and REM) showed that CB1 KO mice spend more time in wake at the expense of NREM (Fig. 10A). Specifically, there was a significant interaction between time of day and genotype nested within photoperiod for the total time spent in wake (HLM, F(12, 51.98) = 2.67, *p* = 0.007) with a main effect of genotype (F(1, 27.13) = 11.30, *p* = 0.002). Pair-wise comparisons revealed a significant increase in the amount of time CB1 KOs spent awake during the first 6 hours of the dark phase (6 to 9 am, F(1, 79.95) = 6.55, *p* = 0.012; 9 to 12 am, F(1, 79.95) = 4.71, *p* = 0.033). An analysis of wake architecture across the circadian cycle found no genotypic differences in either the number of wake bouts or their duration (data not shown). These results indicate that CB1 KO mice exhibit a more active phenotype than *wt* mice.

The increase in wake observed in CB1 KO mice is exclusively due to reduced time spent in NREM sleep (Fig. 10A). There was a significant 3-way interaction (genotype×time nested within photoperiod) for the time spent in NREM (HLM, F(12, 52.071) = 2.11, *p* = 0.032) and a main effect of genotype (F(1, 26.23) = 12.389, *p* = 0.002). Post-hoc analyses revealed a significant reduction in NREM for CB1 KO mice for the first half of the dark photoperiod (6 to 9 am, F(1, 79.69) = 6.43, *p* = 0.013; 9 to 12 am, F(1, 79.69) = 4.84, *p* = 0.031) which is the active period for mice. An analysis of NREM architecture found that CB1 KO mice have reduced duration of NREM bouts (Fig. 10B; two-way repeated measures ANOVA, main effect of genotype, F(1, 10) = 10.55, *p* = 0.009) particularly during the dark photoperiod (pair-wise comparison CB1 KO vs wild-type, t(20) = 3.071, *p* = 0.012). There was no genotypic effect on the number of NREM bouts as evidenced by the lack of a main effect of genotype (F (1, 10) = 2.318, *p* = P = 0.159) or significant interaction between genotype and photoperiod (two-way repeated measures ANOVA, F (1, 10) = 0.5931, *p* = 0.459). These data demonstrate that the reduction in NREM sleep observed in CB1 KO mice was mainly the result of decreased bout duration, suggesting that chronic loss of CB1 destabilizes the NREM.

To gauge the effects of CB1 deletion on slow-wave oscillations, Fast Fourier Transform was performed on the ECoG waveform, and the resulting power spectra corresponding to NREM epochs were compared between genotypes following normalization (see methods). A 3-way interaction was observed for NREM delta power (Fig. 10C; genotype×time nested within photoperiod, HLM, F(12, 65.374) = 3.181, *p* = 0.001), but there was neither a secondary interaction between genotype and photoperiod (F(1, 47.052) = 0.228, *p* = 0.635) nor a main effect of genotype (F(1, 16.161) = 0.85, *p* = 0.775). Similarly, there were no pair-wise differences between genotypes at any of the time points included in the analysis. The observed interaction was due to the large effect of photoperiod (F(1, 47.052) = 33.342, *p*<0.001) that normally modulates delta power across the circadian cycle. Consequently, although the data demonstrate deficits in NREM sleep in CB1 KO mice under basal conditions, these mice generate delta oscillations during NREM sleep.

With respect to REM sleep, genetic deletion of CB1 has only minimal, if any, effect (Fig. 10A). As with wake and NREM, there was also a 3-way interaction for measures of REM sleep (HLM, F(12, 52.398) = 5.21, *p*<0.001), but there was no main effect of genotype (F(1, 26.857) = 0.082, *p* = 0.78). The secondary interaction between genotype and photoperiod (F(1, 29.451) = 8.13, *p* = 0.008) was likely due to an increase in the time CB1 KO mice spent in REM during the first epoch of the photoperiod (F(1, 79.8) = 6.371, *p* = 0.014). Similarly, there were no genotypic effects on the number or duration or REM bouts (data not shown).

### Genetic Deletion of the CNR1 Gene Blunts the Response to Total Sleep Deprivation (TSD)

**Figure 11:**
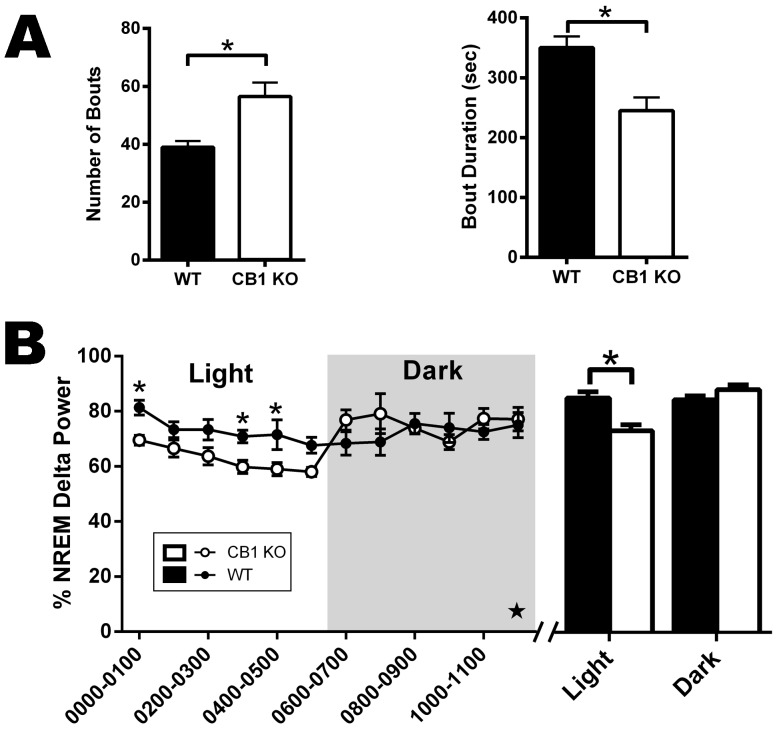
During recovery from total sleep deprivation (TSD), CB1 KO mice exhibit altered sleep architecture and reduced NREM delta power compared to *wt* mice. A, Number and duration of NREM bouts in the last 6± SEM from each genotype (N = 6 for each group). B, NREM delta power during the 12 hr immediately following TSD. Grey background of line graph represents dark photoperiod and white background denotes the light photoperiod. Data in the line graph represent 1 hr bins, and data in the bar graph represent mean ± SEM for 6 hr bins of the specified photoperiod. For all graphs, white bars/circles represent data from CB1 KO mice and black bars/circles represent data from *wt* mice. Significant 3-way interactions between photoperiod, time of day, and genotype are denoted with stars (★). Significant pair-wise comparisons are denoted with asterisks (*).

Although differences in baseline NREM delta power were not observed between genotypes, it is possible that homeostatic mechanisms impose limits on the power of delta oscillations under baseline conditions reducing the likelihood of detecting an effect of CB1 inactivation. In order to test this hypothesis, mice were sleep deprived for the first 6 hr of the light photoperiod (rest phase for mice) and ECoG recordings were obtained for 12 hr immediately following TSD. There were no genotypic differences in the recovery of sleep following TSD (data not shown), but CB1 KO mice had altered sleep architecture in the first 6 hr following TSD (Fig. 11A). KO mice had significantly more NREM bouts than their *wt* littermates (t-test, t(10) = 3.328, *p* = 0.008), but the duration of NREM bouts remained decreased relative to *wt* mice (t-test, t(10) = 3.64, *p* = 0.005). The increased number of short duration bouts likely explains why no difference in sleep recovery time was observed between *wt* and KO mice. Additionally, the instability of the NREM state observed during baseline persists in KO mice after enhancing the homeostatic drive with TSD, suggesting a profound loss of the ability to sustain sleep in the chronic absence of CB1 signaling.

In addition to the differences in sleep architecture observed during sleep recovery, NREM delta power was significantly reduced in KO mice during the first 6 hr immediately following TSD (Fig. 11B). There was a three way interaction between genotype and time nested within photoperiod (HLM, F(20, 77.525) = 1.832, *p* = 0.031) with a secondary interaction between genotype and photoperiod (F(1, 72.68) = 43.664, *p*<0.001). Pair-wise comparisons found significant reductions in delta power during the first (12 to 1 am, F(1, 89.691) = 5.153, *p* = 0.026), fourth (3 to 4 am, F(1, 89.691) = 4.453, *p* = 0.038), and fifth hour (4 to 5 am, F(1, 89.691) = 5.75, *p* = 0.019) of the light photoperiod following TSD. Overall, there was a significant reduction in delta power for KO versus *wt* mice during the entire light photoperiod immediately following TSD when the drive to sleep is highest (F(1, 38.111) = 17.59, *p*<0.001) but not during the dark (F(1, 38.111) = 1.679, *p* = 0.203). These results demonstrate that constitutive loss of CB1 reduces the ability of cortical networks to generate delta frequency oscillations, and this reduction in delta power is correlated with reduced stability of bouts of NREM even when the drive to sleep has been increased.

## Discussion

We found that the EC system modulates up-states in medial PFC PNs, sleep-wake states, and delta frequency oscillations during NREM. Specifically, the exogenous CB1 agonist, WIN, or inhibitors of EC inactivation augmented up-state amplitude and spiking, and these effects were abolished in CB1 KO cultures. The effects of CB1 agonists are consistent with a reduction in GABAergic signaling onto superficial layer PNs. However, NMDA EPSCs were also highly sensitive to WIN demonstrating that CB1's effect on up-states is not simply a result of reduced GABAergic inputs onto PNs. In contrast to the effect of agonists, blockade of CB1 profoundly reduced up-state duration and increased sIPSC frequency on layer II/III PNs suggesting that tonic activation of the CB1 receptor regulates up-state duration. Lastly, up-state duration and spiking were paradoxically enhanced in CB1 KO cultures indicating that while CB1 is not required for up-states, neuroadaptive changes likely occur in the absence of CB1 signaling resulting in a dysregulation of cortical network function. Sleep studies in CB1 KO mice found that genetic deletion of CB1 results in an increase in wake via a specific reduction of NREM sleep maintenance. CB1 KO mice display a reduced ability to maintain NREM even following TSD which is indicative of significant dysfunction in the homeostatic sleep response. Furthermore, the rebound in NREM delta power after TSD is reduced in these mice suggesting that EC signaling contributes to the regulation of slow-oscillations that predominate during this state.

### CB1 modulation of up-states

The present findings are the first to indicate that EC tone exists in the cortex and that tonic activation of CB1 is important for the regulation of network activity. Up-state activity in prefrontal slice cultures was altered by direct activation of CB1 receptors, by modulators of EC inactivation, and by CB1 antagonists. However, slices from KO animals still showed up-state activity demonstrating that CB1 receptors modulate but are not required to generate up-states.

WIN's enhancement of up-state amplitude is consistent with disinhibition of PN's resulting from a CB1-mediated reduction of GABAergic input. Up-states are characterized by robust and persistent glutamatergic transmission that is countered by GABA_A_-mediated shunting inhibition [3], [34]. Reducing GABAergic input via CB1 activation would decrease this inhibition and further depolarize membrane potential. Despite this idea, in the present study WIN inhibited both NMDA and GABA_A_ PSCs in layers II/III and V/VI PNs suggesting that these effects might balance one another. Data from previous studies in the PFC [35], [36] and other regions of cortex [14] also show that EC-CB1 signaling can reduce glutamate transmission suggesting that WIN's effect on up-states is not driven solely by decreased GABAergic signaling. In fact, when IPSC and EPSC effects were compared, WIN produced greater inhibition of EPSCs.

One possible explanation for this apparent paradox is that additional CB1-dependent mechanisms are at work in interneuron populations [32], [37] resulting in a net loss of GABAergic signaling during up-states. Populations of CB1-positive interneurons in the cortex are electrically coupled [38], and this may enhance their susceptibility to CB1-mediated inhibition of intrinsic excitability. In addition, while a small portion of calbindin-positive interneurons express CB1 receptors [39], the majority of CB1-positive GABA terminals are found on perisomatic synapses onto PNs formed by cholecystokinin-positive (CCK+) interneurons [40]. The position of these synapses on PNs makes them particularly well-suited to regulate synchronized patterns of cortical network activity [41], [42] suggesting that disruption of GABAergic signaling from CCK+ neurons may outweigh diminished glutamatergic input due to the sub-cellular compartmentalization of these inhibitory inputs. A comparison of IPSCs between cortical layers revealed that WIN produced greater inhibition of GABA_A_ currents in superficial layer neurons. These data agree with published findings that CB1-mediated inhibition of GABA terminals occurs predominantly in layer II/III PNs [14], [30], [31], [40], [43], [44]. We note that although we monitored up-states in deep-layer neurons in this study, up-states are a network phenomena and essentially all areas of the slice culture show synchronous entry and exit from up-states [10]. In addition, a recent study using an optogenetic approach showed that, in sensorimotor cortex, only a small number of layer V/VI pyramidal neurons need to be activated to initiate up-states and network oscillations in vivo [45] Overall, these data suggest that a CB1-mediated disinhibition of layer II/III PN inputs results in a net excitation of deep layer PNs that is reflected in the alterations in up-state parameters observed in this study.

Up-state amplitude and spiking were also enhanced when inhibitors of 2-AG or AEA metabolism were applied. AEA is also an agonist at TRPV1 receptors [46] that are expressed in cortex [47], but neither the TRPV1 agonist, CAP, nor the TRPV1 antagonist, CPZ, significantly altered up-state parameters as compared to those obtained during control recordings. While both exogenous and endogenous activators of CB1 receptors enhanced up-state amplitude, application of the CB1 antagonists AM251 or AM281 significantly reduced up-state duration and amplitude. As these compounds are actually inverse agonists at the CB1 receptor, their effects may reflect a constitutive activity of CB1 receptors in the slice culture or actions at other G-protein coupled receptors [48]. This seems unlikely, however, as NESS0327, a putative neutral antagonist of CB1 [33], also reduced up-state duration and AM281 had no effect on up-state parameters in CB1 KO cultures. These results suggest that cortical slice cultures generate an EC tone that regulates synaptic activity similar to findings from studies of ECs in the central amygdala [49], hippocampus [50], [51], and spinal cord [52]. Although the mechanism underlying the reduction in up-state duration by CB1 antagonists is not fully known, AM281 also increased the frequency of layer II/III GABA_A_ sIPSCs suggesting that an increased inhibitory drive onto layer II/III PNs may effectively shorten up-states. This proposal appears counter to findings in the literature showing that partial block of GABAergic transmission with bicucculine or SR95531 also reduces up-state duration while simultaneously enhancing spiking [53], [54]. While these differences could reflect methodological variations between these studies involving both species (ferrets, rodents) and brain areas (prefrontal, occipital, entorhinal cortex), it should be noted that application of GABA_A_ antagonists will affect all inhibitory synapses while modulators of EC mediated signaling only affect those synapses that express CB1 receptors. As these include both GABAergic and glutamatergic inputs, the impact of these agents on up-state dynamics could reasonably be expected to be different. While the identity of the EC underlying tonic CB1 signaling in the slice cultures used in this study could not be determined conclusively, treatment with THL, an inhibitor of 2-AG synthesis, had little effect suggesting that AEA may regulate up-state duration. Verifying the involvement of AEA in modulating up-states is more difficult as its synthesis proceeds through a number of distinct pathways. Nonetheless, these data suggest that AEA may be involved in the tonic activation of CB1 receptors and that 2-AG is primarily associated with phasic activation such as that observed during depolarization-induced suppression of inhibition [12], [51].

An important caveat in all of the studies involving long duration (55 min) recordings in the cultures is that we observed fluctuations in our measurement of up-state parameters in sham treated controls. Specifically, duration tended to decrease while amplitude tended to increase relative to baseline values. It is possible that plasticity mechanisms arise from repeatedly evoking up-states, and a recent report has demonstrated that certain forms of striatal inhibitory long-term depression (LTD) are up-state dependent [55]. However, the single pulse stimulation protocol used to evoke neocortical up-states in the present study was delivered at a far lower frequency (0.033 Hz) than that used to induce LTD in previous work, and stimulation was always delivered during the down-state in order to evoke up-states. Therefore, it seems unlikely that plasticity was induced directly as a result of the stimulation protocol used herein. Nevertheless, if plasticity mechanisms emerge as a result of the synaptic activity inherent (and necessary for) the generation of up-states then the results of the studies with CAP, CPZ, and CB1 antagonists could be interpreted as a modulation of these ongoing plasticity mechanisms.

### CB1 is not necessary for the manifestation of up-states

Results from the CB1 antagonist studies suggest that up-states require functional CB1 receptors. Paradoxically, up-state duration and spiking in cultures from CB1 KO mice were greater than those from *wt* cultures. These findings lead to the conclusion that prolonged absence of CB1 signaling induces neuroadaptations that lead to a rebound increase in network activity. The increased excitability of cortical networks observed here is in keeping with other work demonstrating that a loss of CB1 in cortical structures results in a higher susceptibility to seizures [56], [57]. However, it is important to point out that the network activity observed in the present work is not a result of uncontrolled depolarizations characteristic of seizures but reflects normal activity akin to that observed *in vivo* during sleep or anesthesia. Some of the alterations observed in KO cultures could result from aberrant developmental processes as CB1 is associated with axon guidance [58]. It is also possible that the increased duration of up-states in KO cultures could reflect an allostatic process that emerges in response to chronic loss of cortical EC signaling. Future work with conditional knockouts that delete CB1 from specific cell types will be useful in verifying the cellular locus of CB1's effects on up-state parameters.

### Modulation of sleep-wake states via the CB1 receptor

Given the results of increased network activity in CB1 KO cultures, we postulated that CB1 null mutant mice would exhibit reductions in NREM, the sleep stage when up-/down-state transitions are most often observed [1]. The results obtained from the ECoG/sleep studies confirmed this hypothesis, and also suggested that CB1 is involved in regulating deep-stage NREM delta oscillations. Overall, these data are in agreement with studies showing that exogenous CB1 agonists enhance NREM in several mammalian species [59]–[61] and that administration of CB1 antagonists increases wake and reduces NREM sleep [62]–[64]. Furthermore, our results are in agreement with a recent study reporting that deletion of CB1 in subpopulations of forebrain neurons in mice did not affect delta power under baseline conditions [65]. However, in the present study, we also show that chronic loss of CB1 reduces NREM delta rebound following TSD. Sleep deprivation increases the homeostatic drive to engage in NREM sleep and, in control animals, significantly increases the dynamic range of delta power. However, it should be emphasized that the CB1 KO mice used in this study lack functional CB1 expression throughout the body (including the entire cerebrum), and thus, the effects on sleep are unlikely to be limited to CB1's role in the cortex. Furthermore, these mice lack CB1 throughout development, and CB1 has a known role in axon guidance [66] Therefore, the effects on sleep may be attributable to aberrant developmental processes that emerge in the absence of the CB1 receptor. Nevertheless, long-term reduction in CB1 is associated with several neuropsychological disorders that are also associated with sleep disturbances including alcohol dependence [67] and schizophrenia [68], [69].

The findings that CB1 receptors are important in sleep are complemented by studies showing a hypnogenic role for AEA [63], [64], [70]. These data provide one potential mechanism through which the EC system may modulate NREM sleep. Future studies with cell-type specific, targeted deletion of CB1 (particularly in CCK+ interneurons) would be useful in testing some of the hypotheses that emerge from the present study.
